# Distance learning, technological devices, lifestyle and behavior of children and their family during the COVID-19 lockdown in Lombardy: a survey

**DOI:** 10.1186/s13052-021-01156-8

**Published:** 2021-10-11

**Authors:** Marina Picca, Paola Manzoni, Gregorio Paolo Milani, Susanna Mantovani, Claudio Cravidi, Danila Mariani, Angela Mezzopane, Roberto Marinello, Chiara Bove, Paolo Ferri, Marina Macchi, Carlo Agostoni

**Affiliations:** 1 SICuPP-Lombardia: Società Italiana delle Cure Primarie Pediatriche - Lombardia (Italian Primary Care Paediatrics Society-Lombardy), Milan, Italy; 2 Fondazione IRCCS Ca’ Granda - Ospedale Maggiore Policlinico, Pediatric Area, Via della Commenda 9, 20121 Milan, Italy; 3 Department of Clinical Sciences and Community Health, University of Milan, Milan, Italy; 4 Bambini Bicocca S.r.l., University of Milan-Bicocca, Milan, Italy; 5 Department of Human Sciences for Education, University of Milan-Bicocca, Milan, Italy

**Keywords:** COVID-19, Remote working, Distance learning, SARS-CoV-2, Consequences

## Abstract

**Background:**

The COVID-19 pandemic and the subsequent national lockdowns, school closures and distance learning may have had both negative and positive effects on physical and mental health of children.

**Methods:**

A cross-sectional study was conducted on a large group of children between 1 and 10 years old in Lombardy, Italy (*n* = 3392). Their parents filled in a survey answering single or multiple-choice questions about their offspring’s behavior changes (including sleep, dietary habits, emotional disturbances), relationship with siblings, parents and peers, the use of digital technologies, and distance learning experience during the lockdown.

**Results:**

Parents reported lifestyles and emotional alterations during the lockdown. The modifications of family relationships, parents’ remote working, and screen time were associated with sleep, emotional and behavioral modifications. Distance learning was overall considered adequate.

**Conclusions:**

This study reported the most updated data on the effects of COVID-19 pandemic lockdown on children between 1 and 10 years of age in a large sample of Italian schoolchildren. The results of this study point out that pediatricians and authorities should support relationships within families during the COVID-19 pandemic. Parents’ remote working might play an important role for this purpose.

## Background

The COVID-19 pandemic had an impact on everyday life, creating a prolonged period of uncertainty for everyone, including children. To reduce the virus’ spread, several measures have been implemented, including national lockdowns and school closures with subsequent distance learning (DL) through digital classes. According to available evidence, these restrictions have led to temporary social isolation and consequent reduction of cognitive and physical stimuli, with potential negative effects on physical and mental health of children [1–5]. Moreover, it is known that isolation tends to be correlated with higher screen exposure, reduced physical activity and movement, worse dietary habits and sleep disturbances [4–11].

However, only a few studies on the topic are available in the literature and they mainly focused on the negative effects of lockdown on behavioral and psychological functions, such as lower social cognition and emotional development and socio-affective problems [5–9, 12].

The present study aimed to investigate the effects of the COVID-19 lockdown on pre and school-age children in Lombardy (the region with the major burden of COVID-19 in Italy) regarding behaviors and daily life, with special emphasis on distance learning and the use of digital device and.

## Methods

A cross-sectional study (*“Bambini e lockdown: la parola ai genitori” -* “Children and lockdown: the voices of the parents”) was performed between July and August 2020 in Lombardy, Italy, shortly after the spring lockdown period ended.

Data from 3392 children between 1 and 10 years old were collected using an anonymous on-line survey distributed by the SICuPP network (*“Società Italiana delle Cure Primarie Pediatriche”* – “Italian Society of Primary Care”) Lombardy, in collaboration with the University of Milano Bicocca and “Bambini Bicocca” (“Children Bicocca”). Demographic information on the children, including, among other items, the age, gender and number of siblings, was collected. Characteristics of the house, number and type of technological devices available in the family were also investigated. Moreover, parents filled-in single or multiple-choice questions about their offspring’s behavior regarding sleep, nutrition, emotional state, relationship with siblings, parents, and peers, the use of digital technologies, and DL experience.

Regarding their child’s daily habits, parents were asked about possible changes during lockdown in the relationships between children and parents or siblings (“positive”, “negative”, “no relevant change”), sleep (“reduced hours of sleep”, “night awakenings”, “nightmares”, “daytime sleepiness”, “difficulty falling asleep”) and nutritional habits including “modification of appetite” (decreased, not modified, increased) and “consumption of snacks between meals” (decreased, not modified, increased). The occurrence of irritability/tantrums (for children 1–5 years old) or irritability /rage (for children 6–10 years old), attention disturbances (e.g. reduced ability to focus on a task) of children were also asked.

The collected information was used in an anonymous and aggregate form, in compliance with the EU General Data Protection Regulation n. 679/2016 (D.gls. n.196/2003 modified by D.gls. n. 101 del 10.08.2018).

Data are presented as frequency and percentages. The possible relationship between the presence of sleep, attention and mood disturbances (for younger children) or irritability (for older children), (dependent variables), and possibly associated factors were explored by multiple logistic regression analysis. The following independent variables were considered: age, gender, number of siblings, modifications of relationship between parents or between parents and the child during the lockdown, parents’ remote working, presence of an external space in the house, the use of digital devices (> 4 h/day in older and > 2 h/day in younger children), time spent for TV watching. Akaike information criterion was used to select the most statistically relevant variables.

The study was conducted in compliance with all ethical research standards. All parents provide their consent to participate. The entire research process was monitored and approved by the University of Milano Bicocca.

## Results

Data were collected for 3392 children of whom 1688 (49.8%) between 1 and 5 years old (YC) and 1704 (50.2%) between 6 and 10 (OC). Males were 51 and 53% in YC and OC group, respectively. On average, there were two children per family.

Data about daily life and emotion items were partially previously published [13] and the most interesting data are summarized in Table 1.

**Table 1:** Data on sleep, nutrition, and moods disturbances in YC (1–5 years of age) and OC (6–10 years of age) group (13)

	Children 1–5 years %^a^	Children 6–10 years %^a^
**Sleep disturbances**	40	48
**Dietary habits modifications**	40	48
**Mood changes**	61	72
**Irritability/tantrums (YC) - Irritability/rage (OC)**	81	68

^a^ Percentages refer to the number of parents who reported a change in their child during the lockdown(*N* = 1142 in children 1–5 years; *N* = 1177 in children 6–10 years)

Many electronic devices were present in each family: one or often two computers, two or more mobile phones and printers. In the OC group, 64% had access to parents’ devices, 36% used a personal device for DL, 89% of mothers followed their children during DL. Regarding teaching methods, the majority (72%) of teachers used live video-lessons, while a smaller percentage used electronic register or email, 43% used a virtual classroom, 39% recorded video lessons, and 14% digital contents.

**Fig. 1:**
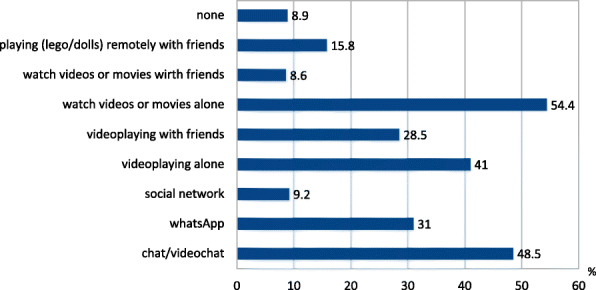
Leisure activities performed by children in OC (6-10 years of age) group

**Fig. 2:**
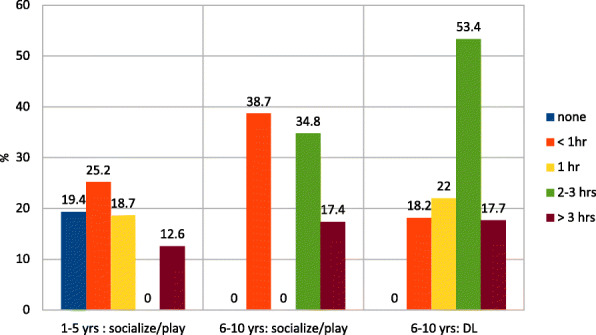
Time (hours) spent using digital devices for socialize, play and distance learning (DL) in YC (1-5 years of age) and OC (6-10 years of age) group

For leisure activities, socializing and play, half of subjects in the OC group used parents’ devices, 24% a personal device, and both 18%. The activities performed are reported in Fig. 1. The time spent using electronic devices for socialize, play and DL is illustrated in Fig. 2. An increase of time for TV watching was reported in 41 and 32% of YC and OC, respectively.

Finally, the results of the multiple logistic regression for sleep, attention and irritability disturbances in OC are given in Tables 2, 3 and 4, respectively. The results of multiple logistic regression for sleep, attention, and moods disturbances are reported in Tables 5, 6 and 7 for YC, respectively.

**Table 2:** Odds ratios (and confidence intervals) of sleep disturbances in children 6–10 years of age during the lockdown period

	***Odds ratio***	***Lower 95% CI***	***Upper 95% CI***	***p-Value***
**Sex (male)**	0.76	0.61	0.97	0.02
**Number of siblings**				
1	1.11	0.82	1.50	0.48
2	0.74	0.51	1.08	0.12
3	0.98	0.60	1.61	0.93
4 or more	0.42	0.12	1.43	0.17
**Relation between parents during lockdown**				
Worsened	1.18	0.88	1.60	0.27
Improved	0.75	0.57	0.98	0.04
**Remote work**				
One parent	0.87	0.66	1.15	0.33
Both parents	0.51	0.38	0.69	< 0.0001
**Time spent for screentime (hours/day)**	1.32	1.04	1.68	0.02
**Time spent for watching television**				
Decreased	1.29	0.79	2.11	0.32
Increased	1.46	1.12	1.90	0.005

**Table 3:** Odds ratios (and confidence intervals) of attention disturbances in children 6–10 years of age during the lockdown period

	***Odds ratio***	***Lower 95% CI***	***Upper 95% CI***	***p-Value***
**Gender (male)**	0.78	0.61	0.99	0.04
**Relation between parents during lockdown**				
Worsened	2.11	1.53	2.92	< 0.0001
Improved	0.87	0.65	1.15	0.3
**Remote work**				
One parent	1.16	0.87	1.57	0.3
Both parents	0.82	0.60	1.13	0.2
**Time spent for screentime (hours/day)**	1.25	0.97	1.61	0.08
**Time spent for watching television**				
Decreased	1.27	0.75	2.15	0.37
Increased	1.32	1.01	1.73	0.04

**Table 4:** Odds ratios (and confidence intervals) of irritability disturbances in children 6–10 years of age during the lockdown period

	***Odds ratio***	***Lower 95% CI***	***Upper 95% CI***	***p-Value***
**Age (years)**	0.92	0.84	1.01	0.09
**Relation between parents during lockdown**				
Worsened	1.98	1.29	3.03	0.002
Improved	0.91	0.67	1.23	0.5
**Relation between parents and the child during lockdown**				
Worsened	7.86	4.83	12.8	< 0.0001
Improved	0.58	0.44	0.78	0.0002

**Table 5:** Odds ratios (and confidence intervals) of sleep disturbances in children 1–5 years of age during the lockdown period

	***Odds ratio***	***Lower 95% CI***	***Upper 95% CI***	***p-Value***
**Age (years)**	0.86	0.79	0.95	0.003
**Relation between parents during lockdown**				
Worsened	1.54	1.12	2.11	0.008
Improved	1.05	0.77	1.42	0.8
**Relation between parents and the child during lockdown**				
Worsened	1.49	1.08	2.06	0.01
Improved	0.91	0.66	1.26	0.58
**Remote work**				
One parent	0.64	0.47	0.86	0.003
Both parents	0.60	0.44	0.83	0.002
**Presence of an external space in the house**				
Balcony	0.57	0.35	0.94	0.03
Courtyard	0.36	0.23	0.58	< 0.0001

**Table 6:** Odds ratios (and confidence intervals) of attention disturbances in children 1–5 years of age during the lockdown period

	***Odds ratio***	***Lower 95% CI***	***Upper 95% CI***	***p-Value***
**Age (years)**	1.69	1.52	1.90	< 0.0001
**Relation between parents during lockdown**				
Worsened	1.30	0.93	1.82	0.1
Improved	0.72	0.52	1.00	0.05
**Relation between parents and the child during lockdown**				
Worsened	2.16	1.54	3.02	< 0.0001
Improved	0.91	0.64	1.28	0.6
**Use of a device for more than 2 h per day**	1.42	1.04	1.94	0.03
**Time spent for watching television**				
Decreased	0.73	0.29	1.87	0.5
Increased	1.43	0.59	3.50	0.4

**Table 7:** Odds ratios (and confidence intervals) of moods disturbances in children 1–5 years of age during the lockdown period

	***Odds ratio***	***Lower 95% CI***	***Upper 95% CI***	***p-Value***
**Relation between parents during lockdown**				
Worsened	1.06	0.67	1.70	0.8
Improved	0.67	0.46	0.97	0.03
**Relation between parents and the child during lockdown**				
Worsened	9.45	4.72	18.9	< 0.0001
Improved	0.76	0.53	1.10	0.1
**Remote work**				
One parent	0.71	0.46	1.09	0.1
Both parents	0.47	0.30	0.72	0.0005
**Time spent for watching television**				
Decreased	0.65	0.24	1.75	0.4
Increased	1.51	0.57	3.96	0.4

## Discussion

To our knowledge, this is the first large-scale Italian study assessing the effects of lockdown on daily life, behaviors, relationships, technological devices, and distance learning experience during the COVID-19 pandemic in children 1–10 years of age.

This study highlights both negative and positive consequences of the lockdown period in children. Overall, it shows an increase in irritability, sleep and dietary habits alterations, attention disturbances and excessive use of digital technologies. However, an improvement in family relationships was also frequently reported.

In OC group, the male gender, the improvement of family relationships and remote working (by both parents) positively influenced sleep, emotional and behavioral disturbances. On the contrary, screen time and an increase in the time spent watching television negatively influenced sleep habit and attention. Finally, the worsening of family relationships was strongly associated with attention disturbances and irritability. In YC group, parents’ remote working and presence of courtyard positively influenced the sleep, while worsened relation between parents was negatively associated with sleep problems. Overall, our data confirm the correlations between family relations, time spent for screen time and sleep [14, 15].

Improved relationships between parents and their children, and parents’ remote working positively influenced mood disturbances. Similarly, attention disturbances were associated with worsened relations between parents and children and by screen time over 2 h.

The data on the role of relationship between parents and children during lockdown support the assumption that children serve as “emotional barometers” for their family and often reflect the level of stress of parents and caregivers [16]. In addition, these data further highlight the association between parental well-being and health of children [16, 17]. The most innovative aspect of this study that deserves further investigations is the possible positive role of parents’ remote working on the well-being of the whole family.

This survey shows that digital technology has been widely used and likely played a crucial role not only for DL but also for social relationships. On the other hand, the literature points out that the use of digital technology for social relationships deserve a continuous monitoring to prevent an excessive use of such devices [18]. This issue is also supported by data previously published on the same group of children that highlighted the parental concern of addiction to devices especially in younger children [13].

Our findings on DL are comparable to those of previous studies conducted in other countries [19–21]. In one study performed in China in 2020 (an online survey given to children, parents, and teachers) the majority of responders reported that the home-schooling style was acceptable [20]. However, teachers were concerned that students’ academic performance and interest would decline, and parents reported an increase in daily screen time and a decrease in outdoor activity. Negative effects of home-schooling were also described in another multi-center study performed in Europe. Most subjects reported detrimental effects of distance learning both for themselves and their offspring, with insufficient support from schools and generally poor quality of teaching [21].

The results of this study could be a stimulus for further investigations to know if positive effects observed in the first lockdown persist after the resumption of schools with a situation of uncertainty and restrictions. Considering their importance on family attitudes and children development, family pediatricians might play a key-role to support such positive aspects [22].

In the future, policymakers should address efforts to support and reinforce the relationship within and between families and community services, particularly pediatricians and schools. In this perspective, it is important to early detect emotional distress both in children and parents and plan interdisciplinary-based support interventions.

The current study has many limitations, including the cross-sectional and single-region design. Moreover, we did not use validated measures and scales. The questionnaire did not consider other potentially influencing factors and the opinions of the children themselves [23]. Finally, information on socioeconomic status of the respondents was not available. On the other hand, the main strengths of this study include the rather large sample size and that it was conducted in Lombardy, a region with a high burden of SARS-CoV-2 infection during the first wave of SARS-CoV-2 infection both on children and adults [24, 25].

## Conclusions

This study reports the most updated data on the effects of COVID-19 pandemic lockdown on Italian children between 1 and 10 years of age, with a focus on changes in daily life, behaviors, social relationships, digital technologies use and distance learning experience. The change of family relationships, parents’ remote working and screen time were associated with sleep, emotional and behavioral modifications. These data show that pediatricians and health authorities should address efforts to support the relationships within families. Parents’ remote working might also play an important, positive role on children health.

## Data Availability

The datasets used and/or analysed during the current study are available from the corresponding author on reasonable request.

## Abbreviations

COVID-19: Coronavirus disease 2019
DL: Distance Learning
SICuPP-Lombardia: Società Italiana delle Cure Primarie Pediatriche Lombardia
